# Gait characteristics in individuals with Parkinson’s disease during 1-minute treadmill walking

**DOI:** 10.7717/peerj.9463

**Published:** 2020-06-29

**Authors:** Byungjoo Noh, Changhong Youm, Myeounggon Lee, Sang-Myung Cheon

**Affiliations:** 1 Department of Health Care and Science, College of Health Sciences, Dong-A University, Busan, Republic of Korea; 2 Biomechanics Laboratory, College of Health Sciences, Dong-A University, Busan, Republic of Korea; 3 Department of Neurology, School of Medicine, Dong-A University, Busan, Republic of Korea

**Keywords:** Parkinson’s, Gait, Aging, Inertial measurement unit, Wearable sensor

## Abstract

**Background:**

No previous study has examined the age-dependent characteristics of gait in individuals between 50 and 79 years simultaneously in healthy individuals and individuals with Parkinson’s disease (PD) over continuous gait cycles. This study aimed to investigate age-related differences in gait characteristics on individuals age ranged 50–79 years, including individuals with PD, during a 1-minute treadmill walking session. Additionally, we aimed to investigate the differences associated with spatiotemporal gait parameters and PD compared in age-matched individuals.

**Methods:**

This study included 26 individuals with PD and 90 participants age ranged 50–79 years. The treadmill walking test at a self-preferred speed was performed for 1 min. The embedded inertial measurement unit sensor in the left and right outsoles-based system was used to collect gait characteristics based on tri-axial acceleration and tri-axial angular velocities.

**Results:**

Participants aged >60 years had a decreased gait speed and shortened stride and step, which may demonstrate a distinct shift in aging (all *p* < 0.005). Individuals with PD showed more of a decrease in variables with a loss of consistency, including gait asymmetry (GA), phase coordination index (PCI) and coefficient of variation (CV) of all variables, than age-matched individuals (all *p* < 0.001). Gait speed, stride and step length, stance phase, variability, GA and PCI were the variables that highly depended on age and PD.

**Discussion:**

Older adults could be considered those older than 60 years of age when gait alterations begin, such as a decreased gait speed as well as shortened stride and step length. On the other hand, a loss of consistency in spatiotemporal parameters and a higher GA and PCI could be used to identify individuals with PD. Thus, the CV of all spatiotemporal parameters, GA and PCI during walking could play an important role and be useful in identifying individuals with PD.

**Conclusion:**

This study provided the notable aging pattern characteristics of gait in individuals >50 years, including individuals with PD. Increasing age after 60 years is associated with deterioration in spatiotemporal parameters of gait during continuous 1-minute treadmill walking. Additionally, GA, PCI and the CV of all variables could be used to identify PD which would be placed after 70 years of age. It may be useful to determine the decline of gait performance in general and among individuals with PD.

## Introduction

Gait refers to locomotion achieved through the movement of human limbs which is regulated by higher level of cognitive functioning and the descending drive from the brainstem to the spinal cord (Hausdorff et al., 2005). The reported spatiotemporal features of gait in aging are a decrease in gait speed, step length and stride length (Menz, Lord & Fitzpatrick, 2003). Of these features, a declined gait performance crucially implies a reduced physical capacity as a result of the aging process (Van Kan, Houles & Vellas, 2012).

Parkinson’s disease (PD) is an age-related neurological disorder. The motor symptoms in individuals with PD are a consequence of the loss of dopaminergic neurons in the substantia nigra (Hausdorff, 2009). Gait disturbances in patients with advanced PD, including spatiotemporal regulation difficulty (Mirelman et al., 2019), reduced gait speed (Lord et al., 2014), longer double support (Do Amaral-Felipe et al., 2020) and cadence (Williams, Peterson & Earhart, 2013), are recognized as contributing diagnostic criteria for PD (Daliri, 2013) and contribute to the risk of falling (Bloem et al., 2004). However, these spatiotemporal parameters are similar to features in old individuals; thus, PD cannot be identified by these variables alone. Therefore, research on gait abilities in PD should consider the assessment of gait symmetry and bilateral coordination, which may have roles in evaluating the altered gait pattern in patients with PD and clinician’s treatment decisions (Yogev et al., 2007; Han et al., 2019). In addition, it is unknown which spatiotemporal parameters of gait are conspicuous traits to aging and PD as well as whether notable patterns are indicative of older age and PD.

Since previous studies could not measure gait consecutively, they were unable to analyze human gait precisely with only a few steps. Previous studies have since recommended the collection of 40 consecutive steps for gait analysis (Rennie et al., 2018). Thus, research on gait with numerous consecutive steps strengthen the reliability of gait-related variables, especially gait asymmetry (GA) and phase coordination index (PCI) (Lee et al., 2020). Moreover, most of the previous studies examined people over the age of 65 years, and no previous study simultaneously examined aging pattern characteristics of gait in individuals aged >50 years, including individuals with PD. Therefore, the primary aim of this study was to investigate age-related differences in gait characteristics on individuals age ranged 50–79 years, including individuals with PD, during 1-minute treadmill walking sessions. The secondary aim was to investigate the differences associated with spatiotemporal gait parameters and PD compared to age-matched individuals. We hypothesized that aging would be associated with a decline in the previously stated spatiotemporal gait parameters. We also suspected that individuals with PD would show a decrease in spatiotemporal gait parameters, which involves GA as a measure of asymmetry, PCI as a measure of bilateral coordination and coefficient of variation (CV) for all variables as a measure of variability.

## Materials and Methods

### Participants

A total of 26 individuals with PD who met the United Kingdom PD Society Brain Bank diagnostic criteria (Gelb, Oliver & Gilman, 1999) and 90 participants in aged 50–59 (50s), 60–69 (60s) and 70–79 (70s) years were recruited (each age group involved 30 participants). In total, 116 volunteers participated in this study. Individuals with PD were recruited from a medical center referral hospital. The inclusion criteria for individuals with PD were as follows (Lee et al., 2018): (a) diagnosis of idiopathic PD, (b) Hoehn and Yahr (H&Y) stages 1–3 (Goetz et al., 2004; Hoehn & Yahr, 1967), (c) a Mini-Mental State Examination score of more than 24 points (Folstein, Folstein & McHugh, 1975) and (d) taking dopaminergic medications (Table 1). Participants were also excluded if they had any history of orthopedic, neurosurgical, or neurological problems within the last 6 months. Participants without PD who matched our participants with PD based on age were additionally included.

**Table 1:** Clinical and demographic characteristics.

Characteristic	Individuals aged in their 50s (*n* = 30)	Individuals aged in their 60s (*n* = 30)	Individuals aged in their 70s (*n* = 30)	Individuals with PD (*n* = 26)
Sex (male/female, *n*)	15/15	16/14	15/15	11/15
Age (years)	54.0 ± 3.0	64.5 ± 2.9	72.5 ± 2.2	66.4 ± 7.3
Height (cm)	163.6 ± 8.6	160.6 ± 9.5	157.6 ± 8.1	158.0 ± 9.0
Body mass (kg)	66.7 ± 12.1	64.5 ± 12.0	63.2 ± 12.4	63.2 ± 10.0
BMI (kg/m^2^)	24.8 ± 2.9	24.8 ± 3.3	26.2 ± 4.7	25.3 ± 3.3
UPDRS total (score)	–	–	–	65.4 ± 15.9
UPDRS part III (score)	–	–	–	7.6 ± 3.3
H&Y stage	–	–	–	2.3 ± 0.4
MMSE score	–	–	–	27.6 ± 2.2
Duration of disease (years)	–	–	–	5.9 ± 3.0
Levodopa dose (mg)	–	–	–	634.8 ± 285.3

**Note:**

Data are presented as means ± standard deviations. n, number; cm, centimeter; m, meter; kg, kilogram; mg, milligram; BMI, body mass index; H&Y, Hoehn and Yahr; MMSE, Mini-Mental State Examination; MOCA, Montreal Cognitive Assessment; PD, Parkinson’s disease; UPDRS, Unified Parkinson’s Disease Rating Scale; 50s, 50–59 years; 60s, 60–69 years; 70s, 70–79 years.

All participants read and signed an informed consent form approved by the institutional review board of Dong-A University (IRB number: 2-104709-AB-N-01-201606-HR-025-04). The study protocol was performed following the tenets of the Declaration of Helsinki.

### Instrumentation

**Figure 1:**
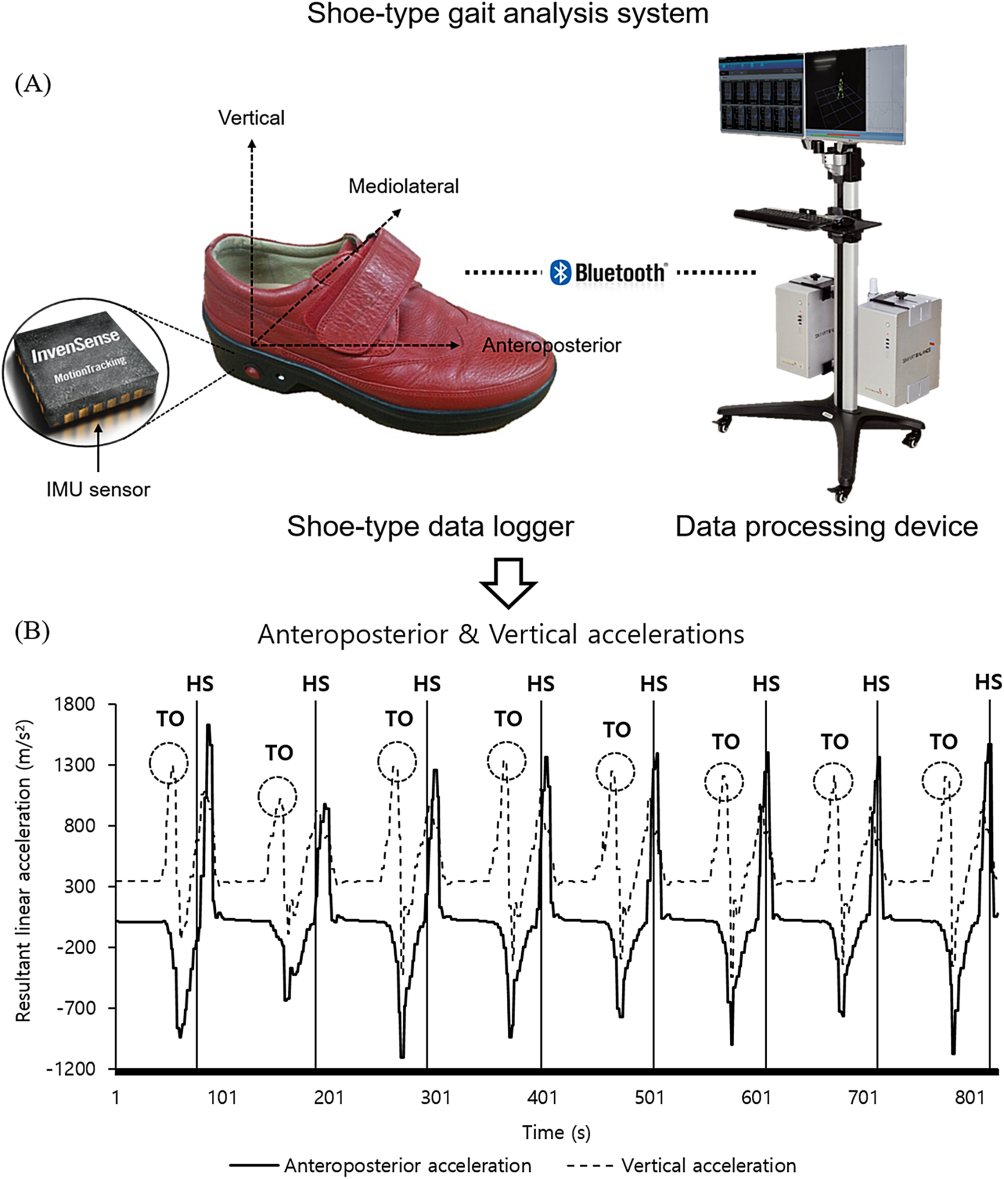
A shoe-type gait analysis system. (A) A shoe-type IMU system composed of shoe-type data loggers with a data acquisition system that was validated with the motion capture system (Lee et al., 2018); (B) gait events are detected in antero-posterior and vertical directions. HS, heel strike; TO, toe off.

Data were collected and analyzed as previously described in Lee et al. (2018; 2020) and Noh et al. (2020). Specifically, the gait analysis system (DynaStab^TM^, JEIOS, South Korea) including shoe-type data loggers (Smart Balance SB-1, JEIOS, South Korea) and a data acquisition system (DynaStab-Spotfire^®^, Tibco Spotfire 7.10) was used. The IMU sensors (IMU-3000^TM^, InvenSense, San Jose, CA, USA) embedded in the left and right outsole of shoe-type data logger. Tri-axial acceleration (up to ± 6 g) and tri-axial angular velocities (up to ± 500° s^−1^) along three orthogonal axes were collected with a sampling frequency of 100 Hz, which was transmitted to a data acquisition system using Bluetooth (Kim et al., 2016; Lee et al., 2018). The local coordinate system of the IMU sensors was established in the antero-posterior, medio-lateral and vertical directions (Fig. 1) (Lee et al., 2018, 2020; Noh et al., 2020). The gait analysis system was validated with the motion capture system (Lee et al., 2018).

### Test procedures

Biometric data (InBody 270, Biospace, Seoul, South Korea), including the body height, weight and body mass index, were recorded prior to the treadmill walking test. Participants with PD were assessed for disease severity using the Unified PD Rating Scale (UPDRS) (Fahn & Elton, 1987). The participants took dopaminergic medication at least 3 h before the treadmill walking session, and the Levodopa equivalent daily dose value was calculated according to Tomlinson, Cullen & Schlossmacher (2010). All PD individuals were in the “on” state at the moment of treadmill walking.

All participants were given verbal or visual instructions to perform treadmill walking at their self-preferred speed for approximately 10 min as a familiarization and warm-up session with a stretching program. They wore the shoe-type embedded IMU sensors that were available in multiple sizes to fit all participants (Lee et al., 2020; Noh et al., 2020).

### Gait performance measures

Gait performance measures were conducted as previously described in Lee et al. (2018; 2020). Specifically, participants were asked to walk at a self-preferred speed on the treadmill (HK–365 Healthkeeper, INFINITY, Seoul, South Korea), which was gradually increased (range of self-preferred speed: 0.14–1.36 m/s). The self-preferred speed was defined as the participant’s comfortable and stable walking condition without any support during the treadmill walking. Gait performance measures were conducted at the self-preferred speed in response to this speed condition to allow more natural gait variability without differences with the fixed speed condition (Sloot, Van Der Krogt & Harlaar, 2014). They walked for approximately 30–60 s from the start of gait, to maintain a steady-state gait, which was a stable gait movement at a constant speed (Lee et al., 2018, 2020). We collected the treadmill walking data for 1 min once they exhibited steady-state gait.

### Data analysis

The data were filtered using a second-order Butterworth low-pass filter with a cutoff frequency of 10 Hz (Kim et al., 2016; Lee et al., 2018, 2020; Noh et al., 2020). For the detection of gait events, heel strikes (HS) and toe-offs (TO) were identified that HS occurred when the linear acceleration along the antero-posterior axis reached its maximum value. Subsequently, TO occurred when the linear acceleration along the vertical axis reached its maximum value (Kim et al., 2016; Lee et al., 2018, 2020; Noh et al., 2020).

Spatiotemporal parameters, such as walking speed, stride length and step length, which were normalized by using them to divide the height of each participant as well as cadence and stance phase, were calculated. Furthermore, we calculated the CV values for all spatiotemporal parameters, which is simply defined as the ratio of the standard deviation for the parameter over the gait cycle (standard deviation/mean × 100), were calculated (Winter, 1991). To assess bilateral coordination, the GA and PCI were calculated based on a previous study (Plotnik, Giladi & Hausdorff, 2007). The GA was defined as the following formula:

$$\rm Gait\; asymmetry\; \left( {GA} \right) = \; 100 \times {\rm \; }\left| {{\rm ln}\left( {\displaystyle{{short\; swing\; time} \over {long\; swing\; time}}} \right)} \right|$$

The PCI values combined the accuracy and consistency of stepping phase generation (Plotnik, Giladi & Hausdorff, 2007). Step time was used to determine the phase (φ). The mean values of the swing time for the left and right leg were calculated. A higher value of the average swing time was used as a reference for the other leg. The relative step timing regarding the stride time (360°), φ_*i*_ (180°), was defined as (Plotnik, Giladi & Hausdorff, 2007):

$${\varphi_i} = 360^\circ \times \displaystyle{{{t_{Si}} - {t_{Li}}} \over {{t_{L\left( {i + 1} \right)}} - {t_{Li}}}}$$

where *t*_*Si*_ and *t*_*Li*_ denote the time of the *i*th heel strike of the legs for the long and short swing times, respectively. The level of accuracy was measured by the mean value of the series of absolute differences (ABS), φ_*i*_−180°, denoted as φ_ABS, a measure used to evaluate the temporal accuracy. The level of consistency of stepping phase generation was also calculated by the CV of φ, denoted as φ_CV (Plotnik, Giladi & Hausdorff, 2007):

$${\rm PCI} = \varphi \_{\rm CV} + {\rm P}\varphi \_{\rm ABS}$$

where

$${\rm P}\varphi \_{\rm ABS} = 100{\rm \; } \times \left( {{{\rm \varphi }_{ABS}}/180} \right)$$

### Statistical analysis

The Shapiro–Wilk test was performed to assess whether the data was normally distributed. The Z-normalization (value–mean/standard deviation) of all variables was performed. One-way analysis of variance (ANOVA) was utilized to detect differences among the participants in each age group, including individuals with PD. Further analyses using Tukey’s honest significant difference test were performed to determine respective differences between individuals aged in their 50s vs. 60s, individuals aged in their 50s vs. 70s, individuals aged in their 50s vs. PD, individuals aged in their 60s vs. 70s, individuals aged in their 60s vs. PD, as well as individuals aged in their 70s vs. PD. The effect size (η^2^) was calculated for the one-way ANOVA’s results. Subsequently, we examined the association of spatiotemporal parameters of gait with aging, including individuals with PD, by multinomial logistic regression. We calculated odds ratios (OR) and 95% confidence intervals (CI) in each spatiotemporal parameter with the participants in their 50s as a reference, which determined the age and PD classifiers for all participants. We also adjusted for all confounders. The analyses were conducted using SPSS for Windows (version 25.0, IBM Corp., Armonk, NY, USA), and *p* < 0.05 was considered statistically significant.

## Results

### Gait characteristics of participants in their 50s–70s and individuals with PD

Table 2 shows the patterns of variables indicating significance between the age groups, including individuals with PD. Walking speed showed a significant main effect for age group. Post hoc analysis indicated that subjects in their 60s and 70s showed significantly slower walking speeds (50s vs. 60s, *p* = 0.002; 50s vs. 70s, *p* < 0.001) than those in their 50s. Regarding stride and step length, subjects in their 70s had a shorter stride length (*p* = 0.001) and step length (*p* = 0.001) than did those in their 50s. There were no significant differences in cadence, stance phase, CV of all spatiotemporal parameters, GA and PCI between age groups.

**Table 2:** Gait characteristics of each age group and individuals with PD.

Variable	Individuals aged in their 50s (*n* = 30)	Individuals aged in their 60s (*n* = 30)	Individuals aged in their 70s (*n* = 30)	Individuals with PD (*n* = 26)	*F*-value (G)	Post-hoc Groups	Effect size (η^2^)
Walking speed (m/s/height)	0.72 ± 0.13	0.59 ± 0.15	0.58 ± 0.12	0.21 ± 0.12	73.841*	a, b, c, e, f	0.312
Cadence (beats/min)	115.13 ± 9.18	109.73 ± 16.91	114.67 ± 10.73	109.50 ± 20.10	1.251	N/A	0.164
Stride length (m/height)	0.75 ± 0.12	0.67 ± 0.15	0.62 ± 0.13	0.24 ± 0.15	74.519*	b, c, e, f	0.332
Step length (m/height)	0.38 ± 0.06	0.33 ± 0.07	0.31 ± 0.06	0.12 ± 0.07	73.618*	b, c, e, f	0.330
Stance phase (%)	61.38 ± 1.26	62.47 ± 2.01	62.66 ± 1.70	62.99 ± 4.72	1.942	N/A	0.254
CV of stride length (%)	1.93 ± 0.93	2.71 ± 1.31	2.72 ± 1.77	5.22 ± 3.15	15.094*	c, e, f	0.253
CV of step length (%)	1.04 ± 0.56	1.65 ± 0.96	1.39 ± 0.81	2.85 ± 1.84	13.283*	c, e, f	0.248
CV of stance phase (%)	2.63 ± 1.29	3.73 ± 1.84	3.68 ± 2.40	8.03 ± 4.77	19.725*	c, e, f	0.255
GA (%)	2.11 ± 2.06	2.79 ± 2.04	2.85 ± 2.48	6.07 ± 4.68	9.817*	c, e, f	0.209
PCI (%)	4.09 ± 2.17	4.56 ± 2.97	5.31 ± 3.12	12.24 ± 6.26	29.239*	c, e, f	0.271

**Notes:**

* A main effect for groups.

Data are presented as means ± standard deviations. Results of post hoc: (a) significance between individuals in their 50s and 60s; (b) significance between individuals in their 50s and 70s; (c) significance between individuals in their 50 s and individuals with PD; (d) significance between individuals in their 60s and 70s; (e) significance between individuals in their 60s and individuals with PD; and (f) significance between individuals in their 70s and individuals with PD. G, main effect for the group; PD, Parkinson’s disease; GA, gait asymmetry; PCI, phase coordination index; CV, coefficient of variation. η^2^, sum of squares between groups/total of squares; N/A, not applicable.

Compared with age-matched individuals, post-hoc test results indicated that individuals with PD had significantly slower walking speeds (50s vs. PD, *p* < 0.001; 60s vs. PD, *p* < 0.001; 70s vs. PD, *p* < 0.001), stride length (50s vs. PD, *p* < 0.001; 60s vs. PD, *p* < 0.001; 70s vs. PD, *p* < 0.001) and step length (50s vs. PD, *p* < 0.001; 60s vs. PD, *p* < 0.001; 70s vs. PD, *p* < 0.001) than age-matched individuals in their 60s and 70s. In contrast, individuals with PD showed a higher CV of stride length (50s vs. PD, *p* < 0.001; 60s vs. PD, *p* < 0.001; 70s vs. PD, *p* < 0.001), CV of step length (50s vs. PD, *p* < 0.001; 60s vs. PD, *p* < 0.001; 70s vs. PD, *p* < 0.001), CV of stance phase (50s vs. PD, *p* < 0.001; 60s vs. PD, *p* < 0.001; 70s vs. PD, *p* < 0.001), GA (50s vs. PD, *p* < 0.001; 60s vs. PD, *p* < 0.001; 70s vs. PD, *p* < 0.001), and PCI (50s vs. PD, *p* < 0.001; 60s vs. PD, *p* < 0.001; 70s vs. PD, *p* < 0.001) than age-matched individuals. There were no significant differences between the groups for cadence and the stance phase.

### Results of multinomial logistic regression model

Table 3 shows statistically significant results from the multinomial logistic regression for all participants. In the multinomial logistic regression models adjusted for confounders, gait-related variables, namely the walking speed, cadence, stride length, step length, stance phase, CVs of the variables (stride length, step length and stance phase), GA and PCI were considered.

**Table 3:** Multinomial logistic regression model for each age group and individuals with PD.

Variable	Individuals aged in their 50s	Individuals aged in their 60s		Individuals aged in their 70s		Individuals with PD	
	OR (ref.)	OR	95% CI	OR	95% CI	OR	95% CI
Walking speed (m/s/height)	1.0	0.23*	[0.09–0.58]	0.18*	[0.07–0.48]	0.00*	[0.00–0.02]
Cadence (beats/min)	1.0	0.68	[0.40–1.16]	0.97	[0.57-1.64	0.671	[0.39–1.16]
Stride length (m/height)	1.0	0.27*	[0.10–0.74]	0.15*	[0.05–0.44]	0.01*	[0.00–0.03]
Step length (m/height)	1.0	0.27*	[0.10–0.74]	0.15*	[0.05–0.44]	0.01*	[0.00–0.03]
Stance phase (%)	1.0	1.72	[0.94–3.16]	1.85*	[1.01–3.38]	2.06*	[1.11–3.82]
CV of stride length (%)	1.0	4.21*	[1.26–14.06]	4.23*	[1.26–14.12]	13.23*	[3.82–45.81]
CV of step length (%)	1.0	5.42*	[1.59–18.42]	4.26*	[1.24–14.63]	14.31*	[4.03–50.79]
CV of stance phase (%)	1.0	5.05*	[1.38–18.52]	4.84*	[1.32–17.77]	27.05*	[6.60–110.87]
GA (%)	1.0	1.58	[0.73–3.40]	1.63	[0.76–3.50]	4.28*	[1.95–9.36]
PCI (%)	1.0	1.70	[0.51–5.67]	2.87	[0.92–8.98]	14.03*	[4.13–47.62]

**Notes:**

* Boldface indicates a statistical significance, *p* < 0.05.

Ref., reference (individuals in their 50s); OR, odds ratio; CI, confidence interval; CV, coefficient of variance; GA, gait asymmetry; PD, Parkinson’s disease; m/s, meter per second; min, minute; %, percent; 50s, 50–59 years; 60s, 60–69 years; 70s, 70–79 years.

Walking speed of subjects in their 60s (OR = 0.23, *p* = 0.002) and 70s (OR = 0.18, *p* = 0.001), as well as individuals with PD (OR = 0.00, *p* < 0.001) relative to subjects in their 50s were associated with age. Stride and step length of subjects in their 60s (stride, OR = 0.27, *p* = 0.011; step, OR = 0.27, *p* = 0.011) and 70s (stride, OR = 0.15, *p* = 0.001; step, OR = 0.15, *p* = 0.001), and individuals with PD (stride, OR = 0.01, *p* < 0.001; step, OR = 0.01, *p* < 0.001) relative to subjects in their 50s were also associated with age. Stance phase of subjects in their 70s (OR = 1.85, *p* = 0.047) and individuals with PD (OR = 2.06, *p* = 0.022) relative to subjects in their 50s was associated with age. The CVs of stride and step length and CV of stance phase in subjects in their 60s (CV of stride, OR = 4.21, *p* = 0.020; CV of step, OR = 5.42, *p* = 0.011) and 70s (CV of stride, OR = 4.23, *p* = 0.019; CV of step, OR = 4.26, *p* = 0.001), and individuals with PD (CV of stride, OR = 13.23, *p* = 0.001; CV of step, OR = 14.31, *p* < 0.001) relative to subjects in their 50s were associated with age. The CV of stance phase in subjects in their 60s (OR = 5.05, *p* = 0.015) and 70s (OR = 4.84, *p* = 0.017), and individuals with PD (OR = 27.05, *p* < 0.001) relative to subjects in their 50s were also associated with age. GA and PCI in individuals with PD (GA, OR = 4.28, *p* < 0.001; PCI, OR = 14.03, *p* < 0.001) relative to subjects in their 50s were associated with age.

## Discussion

This study demonstrates the spatiotemporal features of gait in individuals aged 50–79 with and without PD. The main findings of this study are as follows. (1) Participants exhibited decreased gait speed, shortened stride length and step length with a distinct shift at ages over 60 years. (2) Individuals with PD had lower spatiotemporal parameters of gait, namely slower gait speed and shorter stride and step length than age-matched individuals (in their 60s and 70s). (3) Individuals with PD had a loss of consistency in spatiotemporal parameters and higher GA and PCI compared to age-matched individuals. (4) Gait speed, stride and step length, stance phase, variability, GA and PCI were the variables that highly depended on age and PD. These findings are discussed in detail below.

Previous studies reported that gait speed reduces by up to 16% per decade at 60 years of age (Van Kan, Houles & Vellas, 2012). In our study, decreased gait speed and shortened stride and step length were shown in individuals aged >60 years who had notable changes. Also, our logistic analysis showed age-specific changes in gait speed, stride length and step length in individuals aged >60 years, which were highly dependent on age. Spinal motor neuron apoptosis begins gradually after age 60 years (Cruz-Sanchez et al., 1998) when individuals are approaching their retirement; therefore, older individuals’ physical activity begins to decline. Furthermore, according to analyzed data from the human plasma proteome, aging tends to shift three times during a lifespan at thresholds of 34, 60 and 78 years of age (Lehallier et al., 2019). Although these previous studies could not directly support our findings with respect to gait changes, our data might represent similar patterns to that of physiological changes. Therefore, individuals aged >60 years could be considered the threshold point for when gait alterations, such as a decreased gait speed and shortened stride and step length, begin. In addition, the previous study reported that decreased dynamic gait stability is associated with less steadiness of force output during walking depending on the altered common synaptic input into the motor neuron pools (Negro, Holobar & Farina, 2009). Subsequently, the common synaptic input into the motor neuron pools may be further altered by motor unit synchronization and firing patterns at low intensities (slow gait speed). Therefore, our results may be related to increased stride-to-stride fluctuations during walking with increased foot contact time on the ground, which might be a symptom of instability in old individuals. Thus, these factors would increase the risk of falling in old individuals.

The gait speed, stride length and step length were lower in individuals with PD than in age-matched individuals (aged in their 60s and 70s). The gait characteristics of individuals with PD may be placed after those in their 70s in the current study. Namely, individuals with PD may have a lower level of gait ability than older adults in their 70s. In addition, our logistic analysis showed PD-specific changes in gait speed, stride length, and step length, which were highly dependent on PD. Our findings are similar to those of previous studies that observed decreased gait speed and shortened step and stride length with increased cadence (as the spatiotemporal features of gait) in advanced PD (Do Amaral-Felipe et al., 2020; Lord et al., 2014; Williams, Peterson & Earhart, 2013). Decreased motor ability in individuals with PD is a consequence of a selective loss of dopaminergic neurons, resulting in dopamine deficiency in the substantia nigra, which modulates motor movements as part of the basal ganglia circuitry (Blandini, Armentero & Martignoni, 2008; Hausdorff, 2009; Long-Smith, Sullivan & Nolan, 2009). In addition, a lower level of gait ability may be due to a severe decline in neuromuscular force in individuals with PD; a reduced rate of force development and maximal force have been reported in previous studies that compared individuals with PD and age-matched individuals (Hammond et al., 2017; Rose et al., 2013a). Therefore, muscle power (force × velocity) and muscle strength might both be reduced in individuals with PD, which further affects their walking velocity (Allen et al., 2010).

The present study found that individuals with PD had a higher CV of all spatiotemporal parameters, GA and PCI than age-matched individuals. GA and PCI were associated only with individuals with PD (no association with older adults in their 60s and 70s) in our logistic regression analysis. Indeed, individuals with PD may have a greater fluctuation in gait rhythm than older adults in their 70s. Namely, freezing of gait, which is one of the most debilitating motor symptoms in PD, could be caused by asymmetry and impaired bilateral coordination (Plotnik & Hausdorff, 2008). Previous studies have reported that the brain of individuals with PD have limited capacity to regulate a gait cycle accurately and consistently (Plotnik, Giladi & Hausdorff, 2007), and that all spatiotemporal parameters have increased variability; thus, these individuals lost their ability to generate a steady gait rhythm (Hausdorff et al., 1998). A higher CV of spatiotemporal parameters in individuals with PD may be related to fluctuations in locomotor output (less steadiness) by increased excitability of motor neurons and slow walking speed. Because of greater motor unit loss (Caviness et al., 2002), and disrupted motor unit recruitment patterns (Rose et al., 2013b), type I myofiber grouping (Mu et al., 2012) is observed in individuals with PD, which is the result of motor unit remodeling. Thus, motor unit remodeling in individuals with PD is related to a reduction of force steadiness, which may affect gait automaticity, that is, gait disturbance. Indeed, the CV of all spatiotemporal parameters, GA and PCI might be more sensitive to gait disturbance in individuals with PD.

This study has several strengths. First, our gait performance measures include spatiotemporal parameters with a relatively longer walking time and walkway distance. The available studies have limited the gait performance test to include relatively fewer steps (less than 10 m walkway). However, our study could measure more continuative states for longer durations at a relatively low cost using IMU sensors. Thus, we suggest future research to consider relatively larger steps and longer walking time and distance walkway, which are necessary to determine motor symptoms of PD using these IMU sensors. Second, our gait performance measures also include unique and various variables such as GA, PCI and CV for all variables using IMU sensors. It showed the notable aging pattern characteristics of gait, including individuals with PD for these variables.

The limitations of this study must also be considered. First, we were unable to categorize the participants with PD by motor symptoms such as tremor or freezing of gait with the new freezing of gait questionnaire or postural instability and gait. However, these motor symptoms did not appear during our study. Furthermore, additional gait tasks related to realistic environmental conditions such as turning, changing direction, and walking on irregular surfaces should be considered for individuals with PD. Second, there is no validation literature to measure GA and PCI using the gait analysis system based on an IMU sensor. However, we assumed that GA and PCI could be reliable variables because the validity of the motion capture system was proven by Lee et al. (2018). Finally, we conducted gait performance measures on the treadmill, which may generate different gait patterns than by overground walking. Treadmill walking with a constant speed could contribute to minimizing the stride-to-stride fluctuation in gait timing, whereas overground walking has ongoing fluctuations in gait speed (Frenkel-Toledo et al., 2005). Although, spatiotemporal parameters of gait during treadmill walking are thought to be similar to those during overground walking (Riley et al., 2007). However, this previous study was based on healthy participants. Further studies should assess whether or not treadmill walking is similar to overground walking in individuals with PD.

## Conclusions

We provided notable aging pattern characteristics of gait in individuals age ranged 50-79 years, including individuals with PD. The gait ability, which included various gait variables, deteriorated as a function of age with a threshold of 60 years of age. Further, individuals with PD are associated with higher and less consistent spatiotemporal parameters of gait, which involves GA and PCI, and the CV of all variables. Individuals with PD may be placed after those of 70 years of age in the aging pattern. It may be useful to determine the decline of gait performance and which age group is associated with degeneration of gait ability in general and among individuals with PD. Therefore, our results are considered meaningful to evaluating the altered gait pattern in older adults, including patients with PD and clinician’s treatment decisions.

## Supplemental Information

10.7717/peerj.9463/supp-1 Supplemental Information 1 Levodopa dose of PD patients.

10.7717/peerj.9463/supp-2 Supplemental Information 2 Raw data.

10.7717/peerj.9463/supp-3 Supplemental Information 3 Formula for PCI.

## Additional Information and Declarations

### Data Availability

The following information was supplied regarding data availability: The raw measurements are available in the Supplemental Files.
