# Supplementary material for: Gait characteristics in individuals with Parkinson’s disease during 1-minute treadmill walking
Source: PeerJ. 2020 Jun 29;8:e9463. doi: 10.7717/peerj.9463 (PMC7331622; doi:10.7717/peerj.9463)
Supplement: Supplemental Information 3 [file peerj-08-9463-s003.pdf]

Gait asymmetry was assessed by comparing the swing times performed by one leg with respect to those performed by the other using the following formula:

$$\text{Gait Asymmetry} = 100 \times \left|\ln\left(\frac{Long\ \ swing\ \ time}{Short\ \ swing\ \ time}\right)\right|$$

The PCI value integrates the accuracy and consistency of left-right movement during overground walking. In order to calculate the PCI value, we first calculated the mean of the swing time for both left and right legs, and the higher value of the average swing time was used as a reference for the opposing leg. The relative step timing with respect to the stride time (360°), Φ_i (180°), was determined as follows:

$$\varphi_i = 360° \times \frac{t_{Si} - t_{Li}}{t_{L(i+1)} - t_{Li}} \ .$$

Here, t_{Li} and t_{Si} represent the times of the *i*th HS of the legs for the long and short swing times, respectively, and $t_{L(I+1)} > t_{Si} > t_{Li}$. In order to maintain symmetry during overground walking, the Φ_i value should be approximately 180°. Therefore, the mean value of the series of absolute differences between Φ_i and 180° (Φ_{ABS}) was calculated to evaluate the overall accuracy for each walking speed, where

$$\varphi_{ABS} = \frac{1}{N}\sum_{i=1}^{N}|\varphi_i - 180°| \ .$$

Furthermore, we also calculated the coefficient of variance (CV) of Φ (CV_Φ) in order to assess the level of consistency during the overground walking test. The PCI value was calculated as the combined CV_Φ and percentage Φ_{ABS} ($\Phi_{ABS}/180 * 100$), which represents the accuracy and consistency during overground walking. This PCI definition is expressed as

$$\text{PCI} = CV_\varphi + \quad \text{Percentage}$$

φ_{ABS}. (3)

Reference

1. Plotnik M, Giladi N, Hausdorff JM (2007) A new measure for quantifying the bilateral coordination of human gait: effects of aging and Parkinson’s disease. Exp Brain Res. 181:561-70.
